# Engineering of Humanized Antibodies Against Human Interleukin 5 Receptor Alpha Subunit That Cause Potent Antibody-Dependent Cell-Mediated Cytotoxicity

**DOI:** 10.3389/fimmu.2020.593748

**Published:** 2021-01-08

**Authors:** Jung-Eun Kim, Dong-Hyun Lee, Keunok Jung, Eun-Ji Kim, Youngwoo Choi, Hae-Sim Park, Yong-Sung Kim

**Affiliations:** ^1^Department of Molecular Science and Technology, Ajou University, Suwon, South Korea; ^2^Department of Allergy and Clinical Immunology, Ajou University Medical School, Suwon, South Korea

**Keywords:** IL-5, IL-5 receptor, eosinophil, antagonistic antibody, severe asthma, antibody-dependent cell-mediated cytotoxicity, antibody engineering

## Abstract

Patients with severe eosinophilic asthma (SEA; characterized by persistent eosinophilia in blood and airway tissues) experience frequent asthma exacerbations with poor clinical outcomes. Interleukin 5 (IL-5) and IL-5 receptor alpha subunit (IL-5α) play key roles in eosinophilia maintenance, and relevant therapeutic strategies include the development of antibodies (Abs) against IL-5 or IL-5α to control eosinophilia. Benralizumab, an anti–IL-5α Ab that depletes eosinophils mainly *via* Ab-dependent cell-mediated cytotoxicity and through blockage of IL-5 function on eosinophils, has been clinically approved for patients with SEA. Here, we report engineering of a new humanized anti–IL-5Rα Ab with potent biological activity. We first raised murine Abs against human IL-5Rα, humanized a leading murine Ab, and then further engineered the humanized Abs to enhance their affinity for IL-5Rα using the yeast surface display technology. The finally engineered version of the Ab, 5R65.7, with affinity (*K*_D_ ≈ 4.64 nM) stronger than that of a clinically relevant benralizumab analogue (*K*_D_ ≈ 26.8 nM) showed improved neutralizing activity toward IL-5–dependent cell proliferation in a reporter cell system. Domain level Ab epitope mapping revealed that 5R65.7 recognizes membrane-proximal domain 3 of IL-5Rα, distinct from domain I epitope of the benralizumab analogue. In *ex vivo* assays with peripheral eosinophils from patients with SEA and healthy donors, 5R65.7 manifested more potent biological activities than the benralizumab analogue did, including inhibition of IL-5–dependent proliferation of eosinophils and induction of eosinophil apoptosis through autologous natural-killer-cell–mediated Ab-dependent cell-mediated cytotoxicity. Our study provides a potent anti–IL-5Rα Ab, 5R65.7, which is worthy of further testing in preclinical and clinical trials against SEA as a potential alternative to the current therapeutic arsenal.

## Introduction

Eosinophils are among the main effector immune cells that induce type 2 immune responses associated with the pathogenesis of severe asthma because approximately half of patients with asthma show eosinophilic inflammation (1). The number of accumulated eosinophils in the blood and in airway tissues corresponds to the severity and exacerbations of severe eosinophilic asthma (SEA) (2). Activated eosinophils produce cationic proteins, cytokines, reactive oxygen species, and other substances, which collectively can damage surrounding cells and induce bronchoconstriction and mucus hypersecretion, thereby enhancing type 2/eosinophilic airway inflammation (3). Type 2 helper T cells (T_H_2s) and group 2 innate lymphoid cells (ILC2s) play key roles in the induction of allergic and nonallergic eosinophilic asthma, respectively, by producing a panel of type 2 cytokines, such as interleukin-4 (IL-4), IL-5, and/or IL-13, which are important for the initiation and perpetuation of eosinophilic asthma (4). Among the type 2 cytokines, IL-5 plays the most crucial role in the maturation, proliferation, prolonged survival, and degranulation of eosinophils (2). Hence, antagonists of the activity of IL-5 and its receptor have been developed for the treatment of eosinophilic asthma (5).

IL-5 is a homodimer produced by multiple cell types such as T_H_2 cells, mast cells, and eosinophils. The homodimeric IL-5 binds to a heterodimeric receptor composed of an IL-5–specific subunit called IL-5 receptor alpha (IL-5Rα, also known as CD125) and the common β subunit (βc, also known as CD131) responsible for intracellular signal transduction (6). In humans, IL-5Rα is expressed exclusively on eosinophils and basophils (7) with much higher expression on the former (8). Accordingly, biological effects of the IL-5–IL-5Rα signaling axis are best characterized mainly in the context of eosinophils.

For the treatment of SEA by targeting the IL-5–IL-5Rα signaling axis, recently, three antibodies (Abs), i.e., two anti–IL-5 Abs [mepolizumab (Nucala™) and reslizumab (Cinqair™)] and one anti–IL-5Rα Ab [benralizumab (Fasenra™)], have been clinically approved (5). The two anti–IL-5 Abs specifically interact with IL-5 to neutralize its biological effects on eosinophils by blocking IL-5 from binding to IL-5Rα, thereby indirectly reducing the levels and activation status of eosinophils (5). On the other hand, benralizumab (humanized afucosylated IgG1κ), directed against cell surface–expressed IL-5Rα, depletes eosinophils and basophils by a double mechanism of action: 1) blocking IL-5Rα *via* its Fab portion to prevent IL-5–dependent signaling and 2) inducing Ab-dependent cellular cytotoxicity (ADCC) against IL-5Rα–expressing eosinophils/basophils *via* interactions of its Fc domain with Fcγ receptor IIIa (FcγRIIIa) expressed on natural killer (NK) cells and/or macrophages (9). The enhanced capability of benralizumab to induce ADCC is a consequence of the absence of core fucose on the Fc *N*-glycan; this feature enhances the affinity of its Fc for FcγRIIIa on NK cells and macrophages as compared to the fucosylated parent Ab (8, 10). Up to now, only benralizumab has been marketed as an IL-5Rα antagonist with indication for SEA (11, 12). Despite its well-established clinical efficacy, a subpopulation of asthmatic patients does not respond satisfactorily to benralizumab (13), thus prompting the development of an alternative anti–IL-5Rα Ab with better clinical outcomes.

In the present study, we first generated murine Abs against the extracellular domain of human IL-5Rα and then developed a humanized IgG1κ Ab, dubbed 5R65.7, with strong affinity for IL-5Rα that recognizes epitopes on IL-5Rα that are distinct from those for a benralizumab analogue. Compared with the benralizumab analogue, the 5R65.7 Ab exerted more potent inhibitory activity on IL-5–dependent eosinophil proliferation and NK cell–mediated ADCC against eosinophils from peripheral-blood samples of healthy controls and patients with SEA in *ex vivo* assays. Our work offers a potent anti–IL-5Rα Ab, 5R65.7, as a possible alternative to the current therapeutic arsenal for SEA.

## Materials and Methods

### Isolation of Murine Abs Against Human IL-5Rα

Female Balb/c mice were immunized with a mixture of a purified soluble antigen (100 µg; the extracellular domain of IL-5Rα, residues 21–333), dubbed sIL-5Rα, and Complete Freund’s adjuvant. Two weeks after the administration, 100 µg of sIL-5Rα was administered once a week three times total. Mice that developed positive serum titers for the antigen were chosen for hybridoma fusion. Splenocytes that included Ab-producing B cells from the titer-positive mice were fused with a myeloma cell line (Sp2/0Ag14), and the resulting hybridoma cells were cultured in the 1× HAT culture medium to allow only hybridoma cells to grow. The immunization and hybridoma fusion were performed by A-frontier Inc. (Seoul, Korea). Hybridoma supernatants were screened for binding to sIL-5Rα using ELISA, and cell surface–expressed IL-5Rα on transiently IL-5Rα–expressing HEK293T cells were determined by flow cytometry. Four out of 470 supernatants with relatively strong binding activity for IL-5Rα were isotyped using the Rapid Mouse Ab Isotyping Kit (Pierce, 37503). The four Ab supernatants with heavy chain subclasses of IgG1, IgG2a, or IgG2b were selected for further expansion and purification. To clone the variable domains of heavy chain (VH) and light chain (VL) genes of the m2B7 murine Ab, total RNA was isolated from the hybridoma cells to generate first-strand cDNA fragments by PCR using oligo (dT) primers and Superscript III reverse transcriptase (Invitrogen, #18080093). The cDNA fragments carrying the VH and VL genes of m2B7 were obtained by PCR using the Heavy Primers and Light Primer Mix (GE Healthcare), respectively (14).

### Expression and Purification of Abs and Proteins

The cDNA fragment containing the human *IL-5Rα* gene (amino acid residues 1–420) was kindly provided by Prof. Soo-Hyun Kim (Konkuk University, Korea). To prepare the soluble antigen of sIL-5Rα, the corresponding gene was subcloned in-frame into the pSecTag2A vector to express sIL-5Rα fused with a C-terminal Avi-6× His tag. For IL-5-mFc expression, the human *IL-5* gene (amino acid residues 20–134) (Sino biological Inc., HG15673-G) was subcloned in-frame into the pcDNA3.4 vector to be expressed with a fused C-terminus of mouse IgG2a Fc (hinge-CH2-CH3) (15). The sequences of VH and VL of benralizumab (DrugBank Accession No. DB12023) and bevacizumab (DrugBank Accession No. DB00112) were prepared by DNA synthesis (Bioneer Inc., Daejeon, Korea). The isolated and engineered anti–IL-5Rα Ab clones, the benralizumab analogue, and bevacizumab analogue were reformatted into the human IgG1 isotype through subcloning of respective VH and VL genes into the modified pcDNA 3.4 heavy chain vector (Invitrogen) encoding the human IgG1 constant domain as well as the pcDNA 3.4 light chain vector encoding the kappa constant domain, respectively, as described previously (16–18). The plasmids encoding sIL-5Rα, IL-5-mFc, and Abs were transiently transfected into cultured mammalian human embryonic kidney HEK293F cells in the Freestyle 293F medium (Invitrogen, 12338018) following the standard protocol (16, 17). sIL-5Rα was purified using Ni-NTA resin (GE Healthcare, 17531801). Abs and the IL5-mFc protein were purified on a Protein-A Agarose Chromatography Column (CaptivA, CA-PRI-0100) (18). Protein concentrations were determined by means of the Bicinchoninic Acid (BCA) Kit (Pierce, 23225) *via* measurement of absorbance at 562 nm. To prepare an Ab-screening antigen probe, purified sIL-5Rα samples were biotinylated using the BirA Biotin-Protein Ligase Standard Reaction Kit (Acidity, Bira500) in accordance with the manufacturer’s instructions (18).

### Cell Cultures

Human TF-1 erythroleukemia cells were a gift from Prof. Tae-Hwe Heo (The Catholic University of Korea, Korea). TF-1 cells were maintained in the RPMI 1640 medium (GE Healthcare, SH30027.01) containing 10% of fetal bovine serum (FBS; Thermo Fisher Scientific), 1% of a penicillin-streptomycin solution (WelGENE), and 1 ng/ml GM-CSF (Peprotech, 300-03). Cell lines were authenticated by DNA short tandem repeat profiling (ABION CRO) in 2017 and were used within 20 passages. The cell lines were routinely screened for mycoplasma contamination (CellSafe). Human eosinophils and NK cells were cultured in the RPMI 1640 medium containing 10% of FBS and 1% of the penicillin-streptomycin solution.

### Establishment of TF-1 Cells Stably Expressing IL-5Rα

The human *IL-5Rα* gene (amino acid residues 1–420) was inserted into lentiviral expression plasmid pLJM (Addgene), thus yielding pLJM-IL-5Rα. The pLJM-IL-5Rα plasmid was cotransfected with a mixture of packaging plasmids (pMDLg/pRRE, pRSV/REV, and pMD2-G; Addgene) into the 293FT producer cell line (Invitrogen) *via* Lipofectamine 3000 (Invitrogen) (18). After 48 h of transfection, the viral particles in the culture supernatant were collected, passed through 0.45-μm filters (Corning, 431220), followed by low-speed centrifugation (3000 *g*, 15 min), and then were concentrated with Centricon Plus-20 (Millipore). Viral titers [transducing units (TU)/ml] were determined by transduction of 293FT cells with serial dilutions of the viral solution and by colony counting after puromycin (Gibco, 10 µg/ml) selection. TF-1 cells were transduced with lentiviral particles (10^7^ TU/ml) in the presence of 6 µg/ml polybrene and then selected in a medium containing puromycin (10 µg/ml) to isolate a cell line stably expressing IL-5Rα, named the TF-1/IL-5Rα cell line.

### A TF-1/IL-5Rα Cell Proliferation Assay

TF-1/IL-5Rα cells (5 × 10^4^/well) were cultured in a 96-well plate in the presence of recombinant human IL-5 (rhIL-5; Peprotech, 200-05) and the indicated Abs at various concentrations for 2 days at 37°C and 5% CO_2_. The proliferation was measured by using the CellTiter-Glo (CTG) assay (Promega, G7570), according to the manufacturer’s protocol (18). The luminescence was measured using a Cytation 3 imaging multimode reader (BioTek).

### Affinity Maturation of Abs

Library generation and affinity maturation of Abs were performed using the yeast surface display (YSD) technology, as described previously (18–20). Affinity maturation of Abs by complementarity-determining region (CDR) mutagenesis was conducted in the single-chain antigen-binding fragment (scFab) format involving a G4S-based 63-amino-acid linker between the light chain and heavy chain (19). In the affinity maturation of hu2B7, the targeted residues in VH-CDR2 (residues 53–58 and 60–61) were randomized with a degenerate codon, NHB (17). In the affinity maturation of 5R65, the targeted residues of VL-CDR3 (residues 91–97) and VH-CDR3 (residues 95–100d) were randomized using hand-mixed spiked oligonucleotides (17). For library construction, each amplified *scFab* gene library (12 μg) and a linearized yeast surface display vector (4 μg) were cotransfected 10 times into the *Saccharomyces cerevisiae* AWY101 strain by a homologous recombination technique on Gene Pulser II (Bio-Rad) (17, 20). The diversity of libraries was determined through plating of serial 10-fold dilutions of the transformed cells onto selective agar plates (15). The hu2B7 library was screened *via* four rounds of fluorescence-activated cell sorting (FACS) on FACS Aria III (BD Biosciences) against biotinylated sIL-5Rα (0.5 μM in round 1, 50 nM in round 2, and 10 nM in rounds 3 and 4). The 5R65 library was screened against biotinylated sIL-5Rα in the presence of an excess amount of nonbiotinylated sIL-5Rα as a competitor under kinetic screening conditions (19, 21), as specified in the text. During FACS, cell surface expression and antigen-binding levels of the scFab library were monitored by indirect double immunofluorescent labeling of the CH1 C-terminal c-Myc tag (anti-c-Myc mouse Ab [9E10], 13–2,500, dilution 1:200)/Alexa 488–conjugated goat anti-mouse IgG Ab (Invitrogen; dilution 1:600) and sIL-5α protein [biotinylated antigen/streptavidin (SA)-phycoerythrin (PE)]. Typically, the top 0.1%–0.3% of target-binding cells were separated. The final sorted cells were plated on a selective medium to isolate and analyze individual clones (17).

### An ELISA

Binding specificity of Abs to antigens was determined by an ELISA. Ninety-six-well plates (Corning) were coated at 25°C for 1 h with sIL-5Rα (50 ng/well) or four antigens [dsDNA (25 ng/well), insulin (125 ng/well), hemocyanin (125 ng/well), and cardiolipin (1,250 ng/well)] (18) and blocked with blocking buffer made of PBST [phosphate-buffered saline (PBS) pH 7.4, 0.1% (v/v) of Tween 20, 2% of (w/v) skim milk]. After three washes with PBST, the wells were probed with 8–200 nM Abs in blocking buffer at 25°C for 1 h. The plates were washed and developed as previously described (19). The bound proteins were detected by the addition of a horse radish peroxidase (HRP)-conjugated anti-human Fc Ab (Invitrogen, 628420; dilution 1:8,000) or an HRP-conjugated anti-mouse Fc Ab (Abcam, ab6789; dilution 1:4,000).

For a competitive ELISA, 96-well plates were coated with the IL-5-mFc protein (100 ng/well). After blocking and washing steps, various concentrations of anti–IL-5Rα Abs (0–1 µM) along with sIL-5Rα (50 nM) were added for competitive binding to the plate-immobilized IL-5-mFc at 25°C for 1 h incubation. After three washes, residual binding levels of the sIL-5Rα protein were detected with an HRP-conjugated anti-His Ab (Sigma, A7058, dilution 1:2,000). The binding data were presented as the percentage of sIL-5Rα bound to IL-5-mFc relative to that without Ab competition. Half-maximal inhibitory concentrations (IC_50_) were estimated by fitting normalized dose–response data to a nonlinear sigmoidal curve in GraphPad Prism 5 software (18).

### Determination of Ab Affinity by Bio-Layer Interferometry

Kinetic binding interactions between Abs and sIL-5Rα were measured using an Octet QKe instrument (ForteBio) (18). All kinetic experiments were conducted at 30°C with orbital shaking at 1,000 rpm in 200 µl in 96-well black flat-bottom plates (VWR International, 82050-784). Each purified Ab was diluted to 1 μg/ml in 1× kinetics buffer (Fertebio, 18–1,105, diluted with PBS, pH 7.4) and was directly immobilized on anti-human IgG Fc capture (AHC) biosensors (ForteBio, 18–5,060) at an approximately 1.0 nm response. After an equilibration step of 300 s, the binding isotherms were monitored by exposure of separate sensors simultaneously to different concentrations of sIL-5Rα. The association of the antigen was measured for 300 s, followed by a dissociation step lasting 600 s. For all experiments, an empty reference sensor without the sIL-5Rα antigen was utilized to take into account nonspecific binding of the analyte to the sensor. Association and dissociation rate constants were calculated by fitting to sensorgrams *via* the 1:1 binding model included in the Octet Data Analysis software, version 11.0 (ForteBio).

### Size-Exclusion Chromatography (SEC) Analysis of Abs

SEC analysis of purified Abs was performed on the Agilent 1100 high performance liquid chromatography (HPLC) system with a Superdex 200 10/300GC (10 mm × 300 mm, GE Healthcare) size-exclusion column (18). 20 μg of an Ab (20 μl, 1 mg/ml) was injected onto the SEC column equilibrated with a mobile phase of PBS buffer (pH 7.4) at a flow rate 0.75 ml min^−1^. Chromatograms were obtained by monitoring absorbance at 280 nm. The molecular mass of Abs was estimated using standard molecular mass marker (alcohol dehydrogenase, 150 kDa, Sigma, A8656; bovine serum albumin, 66 kDa; Sigma, A8531).

### Epitope Mapping of Abs

The extracellular domain of human *IL-5Rα* genes (amino acid residues 21–342) and mouse *IL-5Rα* genes (amino acid residues 18−338) as well as the domain-swapped IL-5Rα variants were respectively subcloned in-frame into yeast display plasmid pYDS-H (20).

To ensure enough distance from the yeast cell surface and conformational flexibility, the IgG1 hinge region (residues 216–236, EU number) was inserted between IL-5Rα and a c-Myc tag to be displayed in the IL-5Rα-hinge-c-Myc-Aga2p format. Furthermore, the Cys residues at positions 226 and 229 of hinge region were substituted with Ser to prevent dimerization by disulfide bonding. Cell surface expression levels of the IL-5Rα variants were determined by immunofluorescent labeling of the C-terminal c-Myc tag with 9E10 (1:200 dilution) and secondary labeling with an Alexa 488–conjugated anti-mouse IgG Ab (Invitrogen, A11001) at 4°C for 20 min. To determine the binding of the anti–IL-5Rα Ab to the IL-5Rα variants, IL-5Rα variant–expressing yeast cells (5 × 10^7^) were incubated with one of the anti–IL-5Rα Abs (100 nM) at 25°C for 30 min. The cells were washed with PBSM (Miltenyi Biotec, 130-091-221) and secondarily labeled with an Alexa 488–conjugated anti-human IgG Ab (Invitrogen, A110013) at 4°C for 20 min. After a quick wash with PBS, the cells were subjected to flow-cytometric analysis.

### Flow Cytometry

Surface expression of IL-5Rα on TF-1/IL-5Rα cells, eosinophils, or neutrophils was determined by flow cytometry after direct immunofluorescent labeling of cells with a PE-conjugated Ab against IL-5Rα (BD Pharmingen, 555902). To identify eosinophils or neutrophils, granulocytes were incubated with an allophycocyanin-conjugated anti–Siglec-8 Ab (Biolegend, 347105) [an Ab against sialic-acid–binding immunoglobulin-like lectin (Siglec) 8] for 30 min at 4°C. The cells and Abs were resuspended in FACS buffer (PBS with 2% of FBS and 2 mM EDTA). After a wash with ice-cold FACS buffer, FACS data were acquired using FACSCalibur (Becton-Dickinson) and analyzed in FlowJo V10 software (Tree Star, San Carlos) (18).

### Isolation of Peripheral Eosinophils and NK Cells

All blood samples from patients with SEA were collected according to a protocol approved by the Institutional Review Board (IRB) of Ajou University Hospital (approval ID: AJIRB-GEN-SMP-13-108). Healthy donor blood samples were collected through a protocol approved by the IRB of Ajou University (approval ID: 201602-HM-001-01) (19). All patients and healthy donors provided written informed consent before the sample collection. Patients with asthma got the asthma diagnosis on the basis of recurrent episodes of wheezing, dyspnea, cough, and sputum production as well as evidence of either airway hyperresponsiveness to methacholine or reversible airway obstruction improved by a short‐acting β2 agonist (22). Among the enrolled patients with eosinophilic asthma, severe asthma was defined according to the definition by the International European Respiratory Society/American Thoracic Society guidelines (23). All patients who were current smokers or had comorbid chronic obstructive pulmonary diseases or other chronic diseases affecting asthma outcomes were excluded. Blood samples were collected into BD Vacutainer^®^ tubes containing an acid citrate dextrose solution (BD Biosciences), were stored at room temperature, and processed within 2 h of collection. These samples were layered on a Ficoll-Paque gradient solution (GE Healthcare, 17-5442-03), followed by centrifugation at 879 g and 20°C for 25 min without brakes. The fraction containing granulocytes and red blood cells was separated and placed in Hank’s balanced salt solution supplemented with 2 mM EDTA and 2% of dextran for 40 min incubation. After the removal of red blood cells by hypotonic lysis, eosinophils were separated from the fraction containing granulocytes with the help of the Eosinophil Isolation Kit (Miltenyi Biotec Inc., 130-092-010) according to the manufacturer’s instructions. The purity of eosinophils was consistently >98%, determined by flow cytometry using APC-labeled anti-Siglec 8 Ab. The viability of eosinophils was >99%, judged by trypan blue exclusion assay. The layer containing peripheral blood mononuclear cells (PBMCs) was washed with PBS and centrifuged at 300 g and 20°C for 10 min. After the removal of red blood cells by hypotonic lysis, NK cells were separated from the cell pellet by means of the NK Cell Isolation Kit (Miltenyi Biotec Inc., 130-092-657) according to the manufacturer’s instructions.

### An Eosinophil Proliferation Assay

Human eosinophils (5 × 10^4^) were cultured in a 96-well plate in the presence of rhIL-5 and various Abs at various concentrations for 2 days at 37°C and 5% CO_2_. The proliferation was measured by the CTG assay.

### An ADCC Assay

Human eosinophils (5 × 10^4^/well) were seeded in 96-well U-bottom tissue culture plates (Nunc, 163320). Purified autologous NK cells were washed with a culture medium and added at 2.5 × 10^5^ cells/well (at a target cell–to–effector cell ratio of 1:5). One of the Abs was added along with 100 pM rhIL-5 as described in the text, and the plates were incubated for 20 h at 37°C and 5% CO_2_ for induction of ADCC. The addition of 100 pM rhIL-5 was due to maintain the basal survival of eosinophils during the assay period. After that, the plates were centrifuged at 300 g for 5 min, and 50 μl of the supernatant was removed from each well for an assay of lactate dehydrogenase to determine any potential ADCC activity of the added Ab. The lactate dehydrogenase assays were performed as follows: to each supernatant, 50 μl of CytoTox96 Assay reagents (Promega, G1780) was added, the plate was covered by an opaque box to protect it from light and was incubated for 30 min at room temperature. At the end of color development, 50 μl of Stop Solution (Promega, G183A) was added, and absorbance was measured at 490 nm. The absorbance value of the culture medium was subtracted as a background. A spontaneous release was defined as absorbance of wells containing only the target cells, and a maximum release was defined as absorbance of wells containing target cells lysed with 10× lysis solution (Promega, G182A). The percentage of ADCC for each Ab was calculated according to the following formula: ADCC (%) = 100 × (A – B)/(C – B), where A is an experimental release, B is a target cell spontaneous release, and C is a target cell maximal release with 10× lysis solution (24).

### Statistical Analysis

These analyses were conducted in GraphPad Prism 8 (GraphPad Software, Inc.). Data are presented as the mean ± SEM for pooled data or the mean ± SD for representative data from at least three independent experiments, unless specified otherwise. Comparisons of data between groups were analyzed for statistical significance by the Mann–Whitney test. One-way or two-way analysis of variance (ANOVA) with the Newman–Keuls *post hoc* test was performed to evaluate the significance of differences. In the statistical tests, no correction was made. Data with *P* values less than 0.05 were considered statistically significant.

## Results

### Immunization and Selection of Murine Anti–IL-5Rα Abs

**Figure 1:**
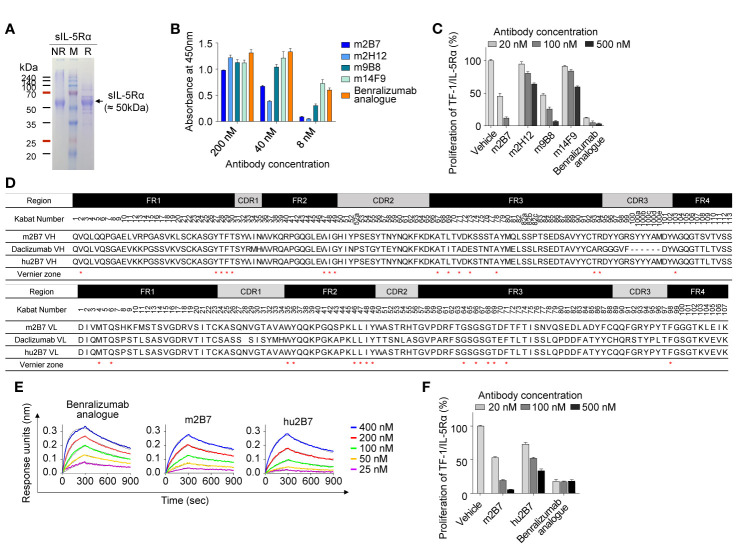
Isolation and humanization of murine Abs against human IL-5Rα. **(A)** SDS-PAGE (12%) analyses of the purified sIL-5Rα proteins (3 µg) under nonreducing (NR) and reducing (R) conditions. Each gel was visualized by staining with Coomassie Brilliant Blue. Molecular masses of the markers (M) are indicated in kilodaltons. **(B)** Analysis of binding the isolated murine anti–IL-5Rα Abs to plate-immobilized sIL-5Rα, as determined by the ELISA. Data are presented as mean ± SD. **(C)** IL-5Rα–blocking activity of murine anti–IL-5Rα Abs, as evidenced by the influence on the proliferation of TF-1/IL-5Rα cells after stimulation with rhIL-5 (80 pM) in the presence of Abs (20, 100, or 500 nM) for 40 h. Data are presented as a percentage (mean ± SD) of the proliferation relative to PBS-treated samples (vehicle). **(D)** Amino acid sequence alignment of the VH and VL regions of murine m2B7, a chosen human acceptor Ab (daclizumab) template, and humanized hu2B7. CDRs and FRs are pointed out with gray and black boxes, respectively. The “Vernier zone residues” described in the main text are marked by asterisks (*). Residues are numbered according to the Kabat numbering system (25). **(E)** Binding isotherms of the immobilized anti–IL-5Rα Abs in relation to the soluble antigen sIL-5Rα, as measured by bio-layer interferometry on the Octet QKe instrument (ForteBio). The concentrations of sIL-5Rα are indicated (colored). The kinetic interaction parameters are listed in **Table 1**. **(F)** IL-5Rα–blocking activity of the indicated Abs, as determined by the effect on the proliferation of TF-1/IL-5Rα cells after stimulation with rhIL-5 (80 pM) in the presence of Abs (20, 100, or 500 nM) for 40 h. Data are presented as a percentage (mean ± SD) of the proliferation relative to PBS-treated samples (vehicle).

To prepare a soluble antigen for mouse immunization, the extracellular domain of IL-5Rα (residues 21–333) containing C-terminal 6×His tags, designated as sIL-5Rα, was expressed in cultured HEK293F cells. The purified protein migrated at ~50 kDa: much larger than its theoretical molecular weight (~39 kDa) under both reducing and nonreducing conditions of SDS-PAGE analysis (**Figure 1A**), indicative of heavy and heterogeneous glycosylation.

For the isolation of Abs against IL-5Rα, female BALB/c mice were immunized with the purified sIL-5Rα, and Ab-expressing hybridomas were generated. Hybridoma supernatants were screened for binding to sIL-5Rα by the ELISA and then for cell surface–expressed IL-5Rα by flow-cytometric analysis of transiently IL-5Rα–expressing HEK293T cells (**Supplementary Figure 1A**). After isotyping for hybridoma supernatants with IL-5Rα-binding activity, four Abs with the mouse IgG subtype Fc domain were selected for further characterization. As a positive-control Ab, we generated the benralizumab analogue of the conventional human IgG1 format, which has VH and VL amino acid sequences identical to those of commercial benralizumab but has the core fucose on the Fc *N*-glycan owing to its expression in HEK293F cells (26). Benralizumab is produced in FUT8 (α1,6-fucosyltransferase) knockout CHO cells to obtain afucosylated Fc *N*-glycan (27). In the ELISA, the four murine Abs and the benralizumab analogue showed concentration-dependent binding to sIL-5Rα with varying magnitude (**Figure 1B**).

To evaluate the antagonistic action of the anti–IL-5Rα Abs on IL-5–dependent cell proliferation, we established a human erythroleukemic TF-1 cell line stably expressing IL-5Rα on the cell surface, designated as TF-1/IL-5Rα cells (**Supplementary Figure 1B**) (28). The TF-1 cells were used because they endogenously express cytokine receptor βc, which can trigger IL-5–dependent cell proliferation signaling after the formation of a complex with exogenous IL-5Rα in TF-1/IL-5Rα cells (28). The isolated murine Abs neutralized the rhIL-5–stimulated proliferation of TF-1/IL-5Rα cells in a dose-dependent manner, though they were much less effective than the benralizumab analogue (10) (**Figure 1C**). Among the four clones, m2B7 showed the highest IL-5–blocking activity, even though the binding to the sIL-5Rα antigen was not the strongest (**Figure 1B**). Hence, we chose m2B7 and cloned the VH and VL genes for the subsequent humanization.

### Humanization of Anti–IL-5Rα m2B7

The framework regions (FRs) of the VH and VL of m2B7 were identified as the mouse germline sequences of IGHV1-61*01 and IGKV6-23*01, respectively (**Figure 1D**). For humanization, the common approach is grafting of six CDRs of murine Abs into the corresponding regions of human germline sequences with maximal homology to the FRs of the murine Ab (10, 15). A BLAST search for the FR sequences of VH and VL against the human germline NCBI IgBlast database yielded the human germline sequences of IGHV1-46*01 and IGKV1-9*01 as the highest-homology sequences, with sequence identities of approximately 66.3% and 65.3%, respectively. Because of the relatively low homology, we blasted the FR sequences of VH and VL of m2B7 against 37 clinically approved therapeutic Abs (The Therapeutic Ab Database; https://tabs.craic.com). Among them, daclizumab, a humanized anti–IL-2Rα Ab (29), featured sequence identities of 75.9% and 71.3% with the VH and VL of m2B7, respectively, excluding the CDRs (**Figure 1D**). Therefore, we selected the FRs of daclizumab as the human acceptor Ab to graft the six CDRs of m2B7 into the corresponding regions of the daclizumab template. Certain residues, referred to as “Vernier zone residues” (30), are located in the β-sheet FR underlying the CDRs often critically affecting CDR loop conformations and thus often need to be maintained with the donor residues to preserve affinity and/or specificity for the target antigen (15, 31). Primary structural analysis of m2B7 and of the acceptor human FR scaffold identified only one Vernier zone residue, ThrH93, in VH (Kabat numbering) (25) that was different between the two sequences. Therefore, the mouse residue ThrH93 in VH was preserved in the humanized Ab (**Figure 1D**). Additionally, 2 murine residues, AspL60 and AspL70, in VL were retained in the humanized Ab owing to their high frequency in the human germline (32). The above procedures generated a humanized Ab dubbed hu2B7 (**Figure 1D**).

The humanized VH and VL sequences of hu2B7 were subcloned into human IgG1 and CL constant domains, respectively (16). Human IgG1 isotype possesses potent Fcγ receptor–mediated effector functions, including ADCC. The plasmids encoding HC and LC of hu2B7 were transiently cotransfected into HEK293F cells to express the hu2B7 Ab in the full-length IgG format. The purified hu2B7 and m2B7 Abs were subjected to bio-layer interferometry analysis to determine the equilibrium dissociation constant (*K*_D_) in relation to sIL-5Rα and showed similar *K*_D_ values in a double-digit nanomolar range: ~47.8 and ~40.2 nM, respectively (**Figure 1E** and **Table 1**). The benralizumab analogue showed somewhat stronger binding affinity (*K*_D_ = 26.8 nM) than those of m2B7 and hu2B7 (**Figure 1E**). Evaluation of the functional activity of hu2B7, i.e., inhibition of rhIL-5–dependent proliferation of TF-1/IL-5Rα cells, revealed dose-dependent IL-5–blocking properties in the following order: benralizumab analogue > m2B7 ˃ hu2B7, manifesting a strong correlation between affinity and the IL-5–neutralizing activity (**Figure 1F**). Given that IL-5 binds to sIL-5Rα with strong affinity (*K*_D_ ≈ 1.9 to 2.4 nM) (6, 33), we reasoned that stronger receptor-binding affinity is required for more potent antagonistic Abs, and therefore, pursued affinity maturation of hu2B7.

**Table 1:** Binding kinetic parameters of the isolated Abs in relation to sIL-5Rα.

	IgG Abs	*K_D_* (nM)	*k_on_* (M^-1^s^-1^)	*k_off_* (s^-1^)	*R*^2^
**Initial screening**	Benralizumab analogue	26.8 ± 0.52	(4.11 ± 0.07)×10^4^	(1.10 ± 0.01)×10^-3^	0.99
	m2B7	40.2 ± 0.84	(2.07 ± 0.04)×10^4^	(8.34 ± 0.09)×10^-4^	0.99
	hu2B7	47.8 ± 1.06	(2.22 ± 0.04)×10^4^	(1.06 ± 0.01)×10^-3^	0.99
**1^st^ affinity maturation**	5R65	14.5 ± 0.29	(2.27 ± 0.03)×10^4^	(3.29 ± 0.05)×10^-4^	0.99
	5R68	24.1 ± 0.47	(2.35 ± 0.03)×10^4^	(5.66 ± 0.07)×10^-4^	0.99
	5R80	16.2 ± 0.59	(2.25 ± 0.05)×10^4^	(3.65 ± 0.10)×10^-4^	0.99
	5R86	11.8 ± 0. 51	(2.54 ± 0.06)×10^4^	(3.00 ± 0.11)×10^-4^	0.99
**2^nd^ affinity maturation**	5R65.7	4.64 ± 0.23	(3.17 ± 0.05)×10^4^	(1.47 ± 0.07)×10^-4^	0.99
	5R65.10	8.25 ± 0.40	(2.44 ± 0.05)×10^4^	(2.01 ± 0.09)×10^-4^	0.99
	5R65.14	8.64 ± 0.35	(2.30 ± 0.04)×10^4^	(1.99 ± 0.07)×10^-4^	0.99
	5R65.18	5.95 ± 0.27	(2.24 ± 0.03)×10^4^	(1.33 ± 0.06)×10^-4^	0.99
	5R65.39	6.13 ± 0.32	(2.80 ± 0.05)×10^4^	(1.71 ± 0.08)×10^-4^	0.99
	5R65.45	7.26 ± 0.36	(2.49 ± 0.05)×10^4^	(1.81 ± 0.08)×10^-4^	0.99

The association rate constant (k_on_), dissociation rate constant (k_off_), equilibrium dissociation constant (K_D_), and an estimate of the goodness of curve fit (R^2^) were calculated in the Octet Data Analysis software, v.11.0 (ForteBio).

Binding kinetic interactions between sIL-5α and immobilized anti–IL-5Rα Abs were measured using bio-layer interferometry.

### Affinity Maturation of hu2B7 to Generate 5R65

**Figure 2:**
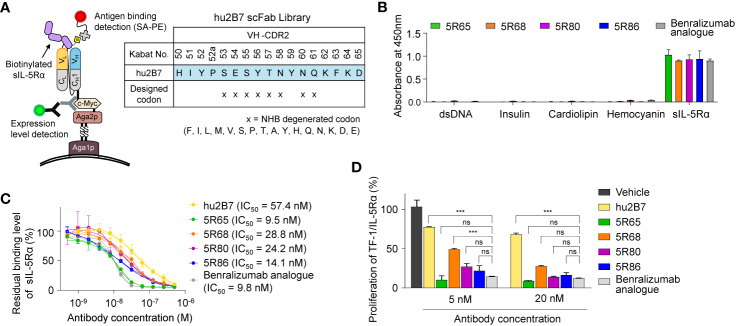
Affinity improvement of hu2B7 to generate a more potent Ab, 5R65. **(A)** The scheme of VH-CDR2 library construction and screening of hu2B7 in the scFab format by the yeast surface display technology. The indicated residues (N) in the VH-CDR2 of hu2B7 were diversified by means of the NHB degenerate codon. **(B)** Evaluation of nonspecific binding of the indicated Abs (100 nM) to four plate-immobilized antigens [dsDNA, insulin, cardiolipin, and hemocyanin] with sIL-5Rα as a control, as determined by ELISAs. Data are depicted as mean ± SD. **(C)** The competitive ELISA showing the percentage of sIL-5Rα (50 nM) bound to plate-immobilized IL-5-mFc in the presence of the indicated Abs (0–0.5 µM) as compared to that without the Ab competitor. Fitted curves were obtained in the GraphPad software. IC_50_ values of the Abs are provided. Each point of the curve represents mean ± SD. **(D)** IL-5Rα–blocking activity of the indicated Abs, as evidenced by the influence on the proliferation of TF-1/IL-5Rα cells after stimulation with rhIL-5 (160 pM) in the presence of Abs (5 or 20 nM) for 40 h. Data are presented as a percentage (mean ± SD) of the proliferation relative to PBS-treated samples (vehicle). Significance was tested by two-way ANOVA followed by the Newman–Keuls *post hoc* test. ***P < 0.001; ns, not significant versus the benralizumab analogue–treated group.

For this purpose, we randomized the second CDR of VH (VH-CDR2) by focusing on the solvent-exposed residues (residues 53–58 and 60–61, Kabat numbering) using a degenerate NHB (ATGC/ACT/TCG) codon. A VH-CDR2–randomized library was generated by the YSD technology in the scFab format in which the C terminus of the heavy chain is linked to the N terminus of the light chain *via* a G4S-based 63-amino-acid linker (**Figure 2A**) (19). The library diversity was ~4 × 10^7^, and sequencing of tens of clones confirmed the fidelity of the library diversity. The yeast library was screened against a biotinylated sIL-5Rα protein in four rounds of FACS with a gradual decrease in the antigen concentration in each subsequent round, thereby yielding four unique good-affinity binders (5R65, 5R68, 5R80, and 5R86; **Supplementary Figure 2A**). The four scFabs were converted into the conventional human IgG1 format and expressed in cultured HEK293F cells.

To evaluate the specificity of the isolated Abs, a multiantigen nonspecificity ELISA was performed by means of four structurally different antigens [double-stranded DNA (dsDNA), insulin, hemocyanin, and cardiolipin] (18). All Abs bound to sIL-5Rα but showed negligible binding activity for the four off-target antigens (**Figure 2B**), thus proving the absence of off-target specificity. Binding affinity of the isolated Abs for sIL-5Rα, as measured by bio-layer interferometry, was stronger (*K*_D_ ≈ 11.8 to 24.1 nM) than that of parental hu2B7 (*K*_D_ ≈ 47.8 nM; **Table 1** and **Supplementary Figure 2B**). Notably, except for 5R68, which showed affinity (*K*_D_ ≈ 24.1 nM) similar to that of the benralizumab analogue (*K*_D_ ≈ 26.8 nM), the others possessed stronger affinity than the benralizumab analogue did (**Table 1**). A competition ELISA revealed that the affinity-improved Abs blocked the binding of sIL-5Rα to human IL-5 fused to the C terminus of mouse IgG2a Fc (IL-5-mFc) in a dose-dependent manner (**Figure 2C** and **Supplementary Figure 2C**). Judging by the IC_50_ values, the affinity-matured Abs possessed improved inhibitory activity than did the parent hu2B7 Ab. Moreover, 5R65 (IC_50_ ≈ 9.5 nM) had similar blocking action on the IL-5–IL-5Rα interaction in comparison with the benralizumab analogue (IC_50_ ≈ 9.8 nM; **Figure 2C**). Compared with the parental hu2B7 Ab, the affinity-improved Abs inhibited the rhIL-5–dependent proliferation of TF-1/IL-5Rα cells more efficiently (**Figure 2D**). Particularly, 5R65 featured the highest antiproliferative activity among the affinity-improved clones, thus showing an activity comparable to that of the benralizumab analogue. Therefore, 5R65 was chosen for another round of affinity maturation.

### Engineering of 5R65 to Generate a More Potent Ab, 5R65.7

**Figure 3:**
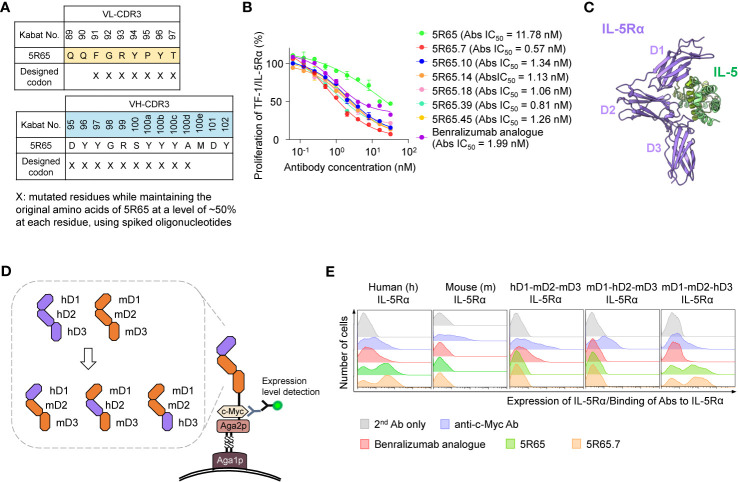
Engineering of 5R65 to generate a more potent Ab, 5R65.7, and domain level epitope mapping of anti–IL-5Rα Abs. **(A)** The scheme of yeast scFab library construction for VL-CDR3 and VH-CDR3 of the 5R65 Ab, where the indicated residues (X) were randomly mutated using spiked oligonucleotides designed to keep the original amino acids at each position of 5R65 at a frequency of approximately 50%. **(B)** IL-5Rα–blocking activity of the indicated anti–IL-5Rα Abs, as determined by the effect on the proliferation of TF-1/IL-5Rα cells after stimulation with rhIL-5 (80 pM) in the presence of Abs (0 to 32 nM) for 40 h. The absolute (Abs) IC_50_ values of the Abs are provided. Each point of the curve represents mean ± SD. **(C)** Overall structure of the human IL-5Rα–IL-5 complex (Protein Data Bank ID: 3QT2), where the three domains (D1, D2, and D3) within the extracellular regions of IL-5Rα are indicated. **(D)** The yeast surface display scheme for domain-swapped IL-5Rα variants, where the three extracellular domains of human IL-5Rα (hD1, hD2, or hD3) were replaced with the corresponding mIL-5Rα domain (mD1, mD2, or mD3). **(E)** Flow-cytometric analysis of yeast cell surface expression levels of the indicated wild-type IL-5Rα and domain-swapped IL-5Rα variants as well as binding activity of the indicated anti–IL-5Rα Abs (100 nM) toward the IL-5Rα variants. Representative histograms from three independent experiments are depicted.

For affinity maturation of 5R65, the VH-CDR3 and VL-CDR3 regions (except for the generally conserved residues M100f, D101, and Y102 in VH-CDR3 and Q89 and Q90 in VL-CDR3) were randomized using hand-mixed spiked oligonucleotides, which were designed to randomly mutate each targeted residue while retaining the parental amino acid sequence at a level of ~50% at each residue (19) to preserve parental residues critically important for IL-5Rα binding (**Figure 3A**). The library was generated in the format of scFab by using the YSD technology. The library with diversity of ~2 × 10^8^ was initially screened against biotinylated IL-5Rα in one round of magnetically activated cell sorting, followed by four rounds of FACS. Notably, FACS was performed under kinetic screening conditions (long dissociation time) to isolate clones with a slow dissociation rate constant (*k*_off_) since *k*_off_ usually dominates in *K*_D_ (= *k*_off_/*k*_on_) particularly in the case of Ab–antigen interactions (21). Briefly, the library (10^8^ cells) was incubated with 5 nM biotinylated sIL-5Rα, washed to remove unbound biotinylated sIL-5Rα, and then incubated with a 2-fold molar excess of nonbiotinylated sIL-5Rα (10 nM) in 1 ml volume at 37°C for 1 h prior to FACS to prevent dissociated biotinylated sIL-5Rα from binding back to yeast cell surface–displayed scFab. In every subsequent round of FACS, competition time was increased by 1 h with washing and the addition of only nonbiotinylated sIL-5Rα (10 nM) every hour. In this way, six unique scFab clones with mutations only in VH-CDR3 (not in VL-CDR3; **Supplementary Figure 3A**) were isolated. The isolated clones were reformatted into human IgG1 form and finally expressed in cultured HEK293F cells.

In the kinetic binding analysis, the isolated Abs showed ~2 to 3-fold stronger affinity for sIL-5Rα (*K*_D_ ≈ 4.64 to 8.64 nM), mainly due to the slower dissociation rates (i.e., lower *k*_off_), as compared with the parental 5R65 Ab (**Table 1** and **Supplementary Figure 3B**). The dose-dependent inhibitory effect of the affinity-matured Abs on rhIL-5–stimulated proliferation of TF-1/IL-5Rα cells was much greater (IC_50_ ≈ 0.57 to 1.34 nM) than that of the parental 5R65 Ab (IC_50_ ≈ 11.8 nM; **Figure 3B**). The relative order in the IL-5α–blocking activity of the Abs was almost same between the assays performed with 80 pM and 160 pM rhIL-5 (**Supplementary Figure 4A**). Notably, the 5R65.7 Ab, which had the strongest affinity (*K*_D_ ≈ 4.64 nM), featured the most potent inhibitory activity (IC_50_ ≈ 0.57 nM) toward the rhIL-5–dependent proliferation, as compared with the engineered Abs and benralizumab analogue (IC_50_ ≈ 1.99 nM; **Figure 3B**). Collectively, these findings suggested that the Abs with stronger affinity due to the slower dissociation rate were able to bind to IL-5Rα for a longer period to prolong the blocking of IL-5 from the receptor binding.

When the purified Abs stored at 4°C for 3 days were subjected to SEC analysis, the original mouse m2B7 and the first humanized hu2B7 Abs showed approximately 6% soluble oligomers (**Supplementary Figure 5A**). However, the humanized and then affinity-matured Abs, 5R65 and 5R65.7, were eluted with a sharp symmetrical peak without detectable oligomers. We further evaluated the stability of the series of engineered anti–IL-5Rα Abs, compared with the original mouse m2B7 Ab, by exposing them to a forced aggregation condition of three freeze-thaw cycles (34). The freeze-thaw cycle stability test revealed no changes in SEC elution profiles for all Abs when comparing the Abs stored at 4°C for 3 days (**Supplementary Figure 5B**), indicating that the affinity-matured Abs, 5R65, and 5R65.7, possess higher colloidal stability than those of the original mouse m2B7 and the first humanized hu2B7 Abs.

### Domain Level Epitope Mapping of Anti–IL-5Rα Abs

In addition to binding affinity, another important determinant of functional activity of antagonistic Abs is the binding epitopes within an antigen. IL-5 binds to the extracellular domain of IL-5Rα by making contacts with all three fibronectin III–like domains of IL-5Rα, with the receptor architecture resembling a wrench (6) (**Figure 3C**). To identify the binding regions of anti–IL-5Rα Abs at the domain level, we generated domain-swapped IL-5Rα extracellular-domain variants, where human IL-5Rα extracellular domains 1 (D1), 2 (D2), and 3 (D3) were replaced with the corresponding murine IL-5Rα (mIL-5Rα) extracellular domain sequences (**Figure 3D**), an approach similar to the one previously reported (8). For the expression of the IL-5Rα extracellular-domain variants, we employed a YSD system to facilitate the expression of stable functional protein domains and to map Ab-binding regions to individual variants (34). All examined Abs—the benralizumab analogue, 5R65, and 5R65.7—bound only to human IL-5Rα without cross-reactivity with mIL-5Rα (**Figure 3E**). The benralizumab analogue bound to the IL-5Rα variant containing human D1 (hD1; i.e., hD1-mD2-mD3) but not to the other variants lacking hD1 (i.e., mD1-hD2-mD3 and mD1-mD2-hD3; **Figure 3E**), confirming the binding epitopes of benralizumab within hD1 of IL-5Rα (8). Notably, both Abs 5R65 and 5R65.7 bound only to the IL-5Rα variant containing hD3 (i.e., mD1-mD2-hD3), i.e., not to the other variants, lacking hD3 (i.e., hD1-mD2-mD3 and mD1-hD2-mD3; **Figure 3E**). This result suggested that the newly engineered anti–IL-5Rα Abs predominantly recognize epitopes within hD3 of IL-5Rα and thus have binding sites distinct from those of benralizumab.

### Isolation and Characterization of Peripheral Eosinophils From Healthy Controls and Patients With Severe Asthma

**Figure 4:**
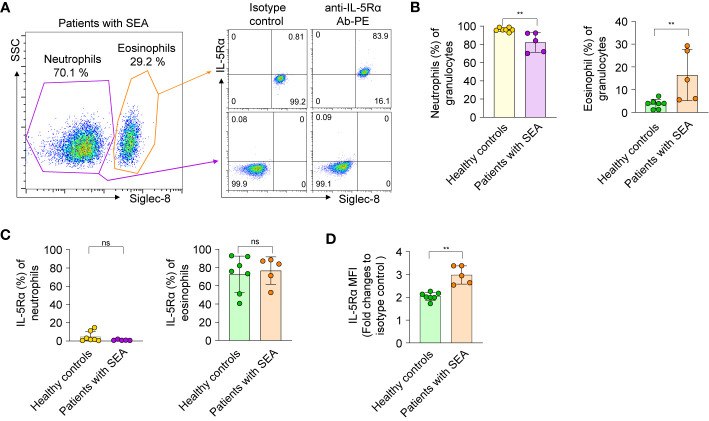
Isolation and characterization of eosinophils and neutrophils from healthy donors and patients with severe asthma. **(A)** *Left panel*: A representative flow-cytometric sorting gate plot of the granulocyte fraction of peripheral blood from a patient with SEA, enriched by Ficoll-Paque separation and then stained with an anti–Siglec-8 Ab. According to side scatter characteristics (SSC) and the cell surface marker Siglec-8, Siglec-8^+^ and Siglec-8^−^ cells were defined as eosinophils and neutrophils, respectively. *Right panel*: Representative dot plots are shown for the cells sorted from the indicated gates and then costained with an allophycocyanin-conjugated Ab against Siglec-8 and a PE-conjugated Ab against IL-5Rα or an isotype control Ab. All data on granulocytes from five patients with SEA and seven healthy controls are given in **Supplementary Figure S4**. **(B)** Proportions of eosinophils and neutrophils among the enriched granulocytes. **(C)** The proportion of cells expressing IL-5Rα on the surface among neutrophils and eosinophils. **(D)** Expression levels of IL-5Rα on eosinophils, as determined by a fold change of mean fluorescence intensity (MFI) of a PE-conjugated anti–IL-5Rα Ab relative to an isotype control Ab. In panels b–d, each circle represents a value obtained from an individual donor. Statistical analysis was performed by the Mann–Whitney test. **P < 0.01, and ns, not significant between the indicated groups.

To examine effects of the anti–IL-5Rα Abs on eosinophils, we isolated circulating eosinophils from human polymorphonuclear cells of five patients with SEA (having blood eosinophil count >300/µl with a mean value of 701 ± 392/µl, n=5) and seven healthy controls (having blood eosinophil count ≤300/µl with a mean value of 220 ± 57/µl, n=7) (**Supplementary Table 1**). With the help of Ficoll-Paque density gradient separation of blood samples, a bottom layer containing granulocytes mainly represented by neutrophils and eosinophils was obtained (35). The granulocyte layer rarely contains basophils and mast cells because basophils partition into the less dense monocyte layer in the Ficoll-Paque solution owing to lower density as compared to eosinophils and neutrophils, and mature mast cells are hardly found in peripheral blood (35). To enrich eosinophils, the isolated granulocytes were further sorted after labeling with an Ab against Siglec-8, the expression of which is restricted to eosinophils, mature mast cells, and basophils (and absent on neutrophils), as described elsewhere (24). The Siglec-8^+^ cells among the granulocytes were defined as eosinophils, whereas the other fraction, Siglec-8^−^ cells, was defined as neutrophils (**Figure 4A** and **Supplementary Figure 6**).

The proportion of circulating eosinophils among granulocytes from healthy donors was approximately 1% to 7% (**Figure 4B**), in line with other reports (36). Of note, the proportion (16.41% ± 11.25%, n = 5) of circulating eosinophils among granulocytes of patients with SEA was 5-fold higher than that (3.67% ± 2.07%, n = 7) of healthy controls (**Figure 4B**). When examined for the expression of IL-5Rα on the cell surface, very few blood neutrophils from patients with SEA and healthy controls expressed IL-5Rα (**Figure 4C**), consistently with another report (37). In contrast, the proportion of IL-5Rα–expressing peripheral eosinophils in patients with SEA was substantial, with variation within 52.6%–88.5%, just as in healthy controls (40.6%–92.9%; **Figure 4C**). Nonetheless, the surface expression levels of IL-5Rα on circulating eosinophils were significantly higher in patients with SEA than in healthy controls (**Figure 4D** and **Supplementary Figure 6**). These data implied that patients with SEA have an increased number of peripheral eosinophils with higher expression of IL-5Rα on the surface, as compared with healthy controls.

### 5R65.7 Exerts More Potent ADCC Against Eosinophils Than the Benralizumab Analogue Does

**Figure 5:**
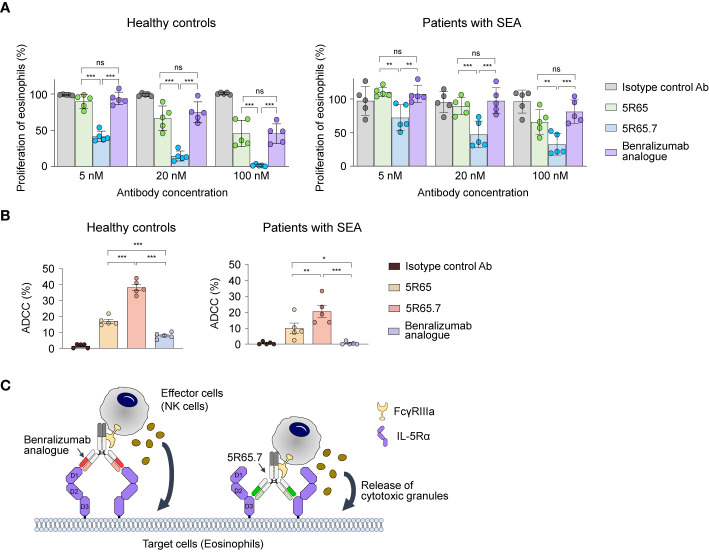
Functional activity of the anti–IL-5Rα Abs against eosinophils from healthy donors and patients with SEA. **(A)** IL-5Rα–blocking activity of the indicated Abs, as evidenced by the influence on the proliferation of eosinophils from healthy controls and patients with SEA after stimulation with rhIL-5 (100 pM) in the presence of Abs (5, 20, or 100 nM) for 40 h. Data are presented as a percentage (mean ± SD, n = 2 or 3) after normalization to the vehicle control (100%). **(B)** The indicated anti–IL-5Rα Ab-mediated ADCC against eosinophils from healthy controls and patients with SEA. Eosinophils were cocultured with NK cells purified from autologous PBMCs at a 1:5 ratio of target cells to effector cells in the presence of rhIL-5 (100 pM) and the indicated Abs (1 μM) for 20 h. Each circle represents a value obtained from an individual donor. Data are presented as percentages (mean ± SD, n = 5). Statistical analysis was performed *via* one-way ANOVA followed by the Newman–Keuls *post hoc* test. *P < 0.05; **P < 0.01; ***P < 0.001 and ns, not significant between the indicated groups. **(C)** A schematic diagram illustrating the difference in ADCC potency between the benralizumab analogue and the 5R65.7 Ab. The benralizumab analogue and 5R65.7 bind to membrane-distal domain D1 and membrane-proximal domain D3 of IL-5Rα, respectively, thereby yielding a corresponding wide or tight cytolytic synapse between an effector NK cell and a target eosinophil.

To determine clinical relevance of the newly developed Ab, we first evaluated the suppressive action of anti–IL-5Rα Abs on IL-5–dependent proliferation of eosinophils purified from human blood. As an isotype control Ab, we generated an anti–vascular endothelial growth factor (VEGF) bevacizumab analogue. Treatment of rhIL-5 at 1, 10, and 100 pM significantly increased the proliferation of eosinophils from healthy controls in proportion to the concentration. However, the difference in the magnitude of the enhanced proliferation according to the concentration was not large, showing only ~20% improvement at 100 pM compared with that at 1 pM (**Supplementary Figure 4B**). Accordingly, we used the highest single concentration (100 pM) of rhIL-5 to evaluate the IL-5α-blocking activity of the Abs. Compared with the control Ab, the anti–IL-5Rα Abs, including the benralizumab analogue, significantly impeded the proliferation of eosinophils from patients with SEA and from healthy controls in a concentration-dependent manner (**Figure 5A**). Notably, the 5R65.7 Ab had the most potent inhibitory impact on the rhIL-5–dependent proliferation of eosinophils, in comparison with the benralizumab analogue and 5R65. The antiproliferative activities of the three anti–IL-5Rα Abs were much greater in relation to eosinophils from healthy controls, as compared with those from patients with SEA, at the same Ab concentration (20 and 100 nM; **Figure 5A**).

Since elevated numbers of eosinophils are mainly associated with the pathogenesis of eosinophilic asthma (38), it is important to reduce eosinophil numbers by inducing their apoptosis *via* ADCC in patients with SEA for better management of asthma symptoms (13). Depletion of eosinophils by NK cell–mediated ADCC is presumed to be the key effector function of benralizumab (11). We evaluated NK cell–mediated ADCC potency of the engineered anti–IL-5Rα Abs by incubating eosinophils—purified from polymorphonuclear cells of healthy donors and patients with SEA—with the respective autologous NK cells isolated from PBMCs at a 1:5 ratio. As IL-5 is crucial for the maintenance of eosinophil survival, the ADCC assay was performed in the presence of IL-5. The benralizumab analogue elicited very weak ADCC by inducing ~10% apoptosis of peripheral eosinophils from healthy controls and negligible ADCC against peripheral eosinophils from patients with SEA (**Figure 5B**), in agreement with another report (8). In contrast, both Abs 5R65 and 5R65.7 substantially induced apoptosis of peripheral eosinophils from healthy donors and from patients with SEA, showing ~1.5- and ~3-fold improved ADCC against peripheral eosinophils from healthy controls as compared with the benralizumab analogue (**Figure 5B**). Although 5R65 and the benralizumab analogue were found to have similar affinity for IL-5Rα, only the 5R65 Ab induced ADCC against the patient-derived eosinophils, suggesting that in addition to binding affinity, binding epitopes of anti–IL-5Rα Abs play a critical role in ADCC. When effector cells, such as NK cells, engage Ab-coated target cells at a short distance, they lyse target cells more efficiently through ADCC because of effective delivery of cytotoxic granules to the target cells (39–41). Therefore, we speculated that Abs 5R65 and 5R65.7, by binding to membrane-proximal domain D3 of IL-5Rα, were more effective at inducing ADCC as compared to the membrane-distal-domain-D1–binding benralizumab analogue (**Figure 5C**). Among the Abs recognizing the same membrane-proximal domain D3 of IL-5Rα, 5R65.7 caused ~2-fold stronger ADCC than the parental 5R65 Ab (**Figure 5B**), suggesting that ~3-fold stronger affinity of 5R65.7 for IL-5Rα (than that of 5R65) contributes to the improved function.

## Discussion

Increased numbers of circulating and airway mucosal eosinophils are a hallmark of SEA and correlate with clinical severity and frequent exacerbations (2, 9, 42). Accordingly, depletion of eosinophils by targeting of IL-5 or its cognate receptor IL-5Rα is a well-validated strategy for the treatment of SEA, as evidenced by the clinically approved Abs: anti–IL-5 mepolizumab and reslizumab as well as anti–IL-5Rα benralizumab (5, 12). Particularly, direct targeting of IL-5Rα on the surface of eosinophils by means of Abs is an attractive therapeutic strategy owing to its rapid and near-complete depletion of peripheral eosinophils and/or basophils *via* induction of apoptosis through ADCC in addition to the direct blocking of IL-5–dependent signaling (9). In the present study, we first raised murine Abs against sIL-5Rα and then performed humanization and affinity maturation by the YSD technology with an appropriate library construction strategy and a suitable screening method, yielding a series of anti–IL-5Rα humanized Abs. These Abs blocked IL-5 from binding to IL-5Rα by recognizing one of the ligand-binding sites on IL-5Rα, particularly, extracellular domain D3, which is distinct from D1 for the benralizumab analogue. The eventually generated 5R65.7 Ab specifically bound to sIL-5Rα with stronger affinity (*K*_D_ ≈ 4.6 nM) and exerted more potent antagonistic action on peripheral eosinophils as compared with the benralizumab analogue. These findings mean that 5R65.7 can be an alternative anti–IL-5Rα Ab targeting activated eosinophils in patients with SEA.

YSD with the eukaryotic quality control system in the protein secretion pathway is a powerful tool to simultaneously optimize stability and/or expression of a targeted Ab in addition to the affinity during the affinity maturation, which is difficult to be achieved by a rational design (43, 44). The affinity-matured 5R65 and 5R65.7 Abs using YSD technology showed higher colloidal stability against 3 freeze-thaw cycles than that of the initial Ab 2B7 (**Supplementary Figure 5**) and comparable expression levels by transient expression in mammalian cells with purification yields of approximately 30–70 mg/L of transiently transfected HEK293F cells to those of commercialized therapeutic Abs (16). However, the purification yields of the affinity-matured Abs, 5R65 (34 ± 4 mg/L, n = 3) and 5R65.7 (61 ± 10 mg/L, n = 3), were not successively improved over that of the initial Ab, hu2B7 (55 ± 8 mg/L, n = 3), implying that the expression of antibody fragment in yeast is not strictly correlated with that of full-length IgG in mammalian cells.

We found that the affinity of the anti–IL-5Rα Abs is a critical determinant of the superior potency of the anti–IL-5Rα Abs. For Ab–antigen interactions, a slower dissociation rate constant, i.e., lower *k*_off_, predominantly determines affinity (*K*_D_ = *k*_off_/*k*_on_) (21). In the present study, we performed affinity maturation of the 5R65 Ab against IL-5Rα under kinetic screening conditions to isolate good-affinity binders (because of the slower dissociation rate), thereby obtaining the final Ab, 5R65.7. The affinity of 5R65.7 was ~3-fold and ~6-fold stronger than that of the parental 5R65 Ab and of the benralizumab analogue mainly owing to the ~2-fold and ~7-fold lower *k*_off_, respectively (**Table 1**), resulting in its superior IL-5–blocking activity and IL-5Rα–opsonized ADCC activity in comparison with the counterparts. Accordingly, the stronger-affinity 5R65.7 Ab may occupy cell surface–expressed antigen IL-5Rα much longer than the weaker-affinity counterparts can; the prolonged retention of the 5R65.7 Ab on the surface of eosinophils leads to its superior ability to block IL-5 binding and more efficient recognition by NK cells to ensure improved ADCC. The correlation between the ability of antagonistic Abs to form slowly dissociating complexes with their cognate membrane receptors and a better ADCC function has also been observed for antitumor Abs against CD19 (45) and HER2/neu (46).

In addition to binding affinity, NK cell–mediated ADCC potency of anti–IL-5Rα Abs seemed to be dependent on the distance from the binding epitope to the target cell membrane. The benralizumab analogue, which recognized membrane-distal domain D1 of IL-5Rα, only weakly induced ADCC toward eosinophils from healthy donors but failed against eosinophils from patients with SEA (8, 10). In contrast, Abs 5R65.7 and 5R65, whose epitope is membrane-proximal domain D3 of IL-5Rα, efficiently elicited ADCC toward eosinophils from patients with SEA and from healthy donors. This finding suggests that the anti–IL-5Rα Abs binding to membrane-proximal domain D3 are more effective in inducing ADCC. This kind of epitope distance effect has been corroborated for NK cell–mediated ADCC by means of an anti-CD173 Ab (39) as well as for CD8^+^ T-cell–mediated ADCC by means of T-cell–engaging bispecific Abs (40, 47). In those studies, the most potent activity was elicited when the Abs recognized membrane-proximal regions rather than distal regions of a given membrane tumor-associated antigen on tumor cells. A prerequisite for ADCC driven by anti–IL-5Rα Abs for NK cells is the formation of a cytolytic synapse between an NK cell and eosinophil, under the influence of the interactions of FcγRIIIa expressed on the NK cell with the Fc of the Ab-opsonizing eosinophil. Our results imply that synapse formation or function is more efficient when an NK cell and eosinophil come closer through bridging of the two cells *via* binding of anti–IL-5Rα Abs to membrane-proximal domain D3 of IL-5Rα, in comparison with the benralizumab analogue binding to membrane-distal domain D1 of IL-5Rα (**Figure 5C**).

The clinically approved version of benralizumab, lacking the fucose sugar on Fc *N*-glycan, has 5- to 50-fold stronger affinity for FcγRIIIa, thereby eliciting more than 1,000-fold enhanced ADCC relative to the wild-type fucosylated counterpart, although its binding affinity for IL-5Rα is indistinguishable from that of the wild-type (8). In our study, all Abs were prepared by HEK293F expression such that they were fucosylated (26), including the benralizumab analogue. We can hypothesize that if we prepare an afucosylated version of the 5R65.7 Ab by the production in FUT8 knockout CHO cells (27), the resultant Ab will be even more superior to benralizumab for the treatment of patients with SEA because its ADCC is enhanced through the formation of a tighter cytolytic synapse (**Figure 5C**).

Since eosinophils serve as a host defender against parasitic, bacterial, and viral infections, eosinophil-depleting therapies, including anti–IL-5Rα Ab treatment, raise the potential risk of increased infections. In a recent phase III clinical trial with benralizumab in patients with SEA, the overall incidence and type of infection adverse effects were similar between benralizumab- and placebo-treated patients and remained stable through the extended treatment period (48), suggesting no apparent association between benralizumab treatment and increased risk of infections. Nonetheless, the potential parasitic infection risks of eosinophil-depleting/lowering therapy should be carefully evaluated in the clinic and real world.

In conclusion, we offer an anti–IL-5Rα humanized Ab, 5R65.7, which more potently inhibits IL-5–dependent eosinophil proliferation and induces NK cell–mediated ADCC against eosinophils from patients with severe asthma, as compared with a benralizumab analogue. The stronger affinity and the membrane-proximal binding epitope of the 5R65.7 Ab are crucial for the superior biological activity in comparison with the benralizumab analogue. These observations suggest that the development of an anti–IL-5Rα Ab for depletion of eosinophils will benefit from targeting of membrane-proximal domain D3 and from the use of a more slowly dissociating good-affinity binder. In this context, it would be worthy of further assessing the efficacy of 5R65.7 Ab in reducing blood and tissue eosinophilia in patients with SEA (blood eosinophil count >300/µl) as well as in patients with refractory hypereosinophilic syndromes (blood eosinophil count >1,500/µl), while comparing with benralizumab. One of the limitations of Ab therapeutics in chronic immunological diseases is the production of an anti-drug Ab due to repeated administration of the same drug. Given that for the targeting of IL-5Rα, only benralizumab has been clinically approved so far, alternative Abs are needed to give physicians the option of switching between anti–IL-5Rα Abs in the management of treatment-refractory SEA and other eosinophilia-related diseases. Finally, the 5R65.7 Ab is a promising candidate for further testing in preclinical and clinical trials against SEA.

## Data Availability Statement

The datasets presented in this study can be found in online repositories. The names of the repository/repositories and accession number(s) can be found in the article/**Supplementary Material**.

## Ethics Statement

The studies involving human participants were reviewed and approved by Institutional Review Board (IRB) of Ajou University Hospital (approval ID: AJIRB-GEN-SMP-13-108) and Ajou University (approval ID: 201602-M-001-01). The patients/participants provided their written informed consent to participate in this study.

## Author Contributions

J-EK screened and engineered the antibodies against IL-5Rα. D-HL, KJ, and YC performed the cell-based assays. E-JK purified the proteins and antibodies. H-SP provided the blood samples of patients and healthy donors. Y-SK, J-EK, and H-SP conceived and designed the experiments. Y-SK and H-SP supervised the project. Y-SK and J-EK wrote the manuscript with input from all the coauthors. All authors contributed to the article and approved the submitted version.

## Funding

This work was supported by a grant of the Korea Health Technology R&D Project (HI16C0992 to Y-SK and H-SP) through the Korea Health Industry Development Institute (KHIDI), funded by the Ministry of Health & Welfare, and a grant from the Priority Research Center Program (2019R1A6A1A11051471 to Y-SK) through the National Research Foundation of Korea (NRF), funded by the Ministry of Science, ICT & Future Planning, Republic of Korea.

## Conflict of Interest

The authors declare that the research was conducted in the absence of any commercial or financial relationships that could be construed as a potential conflict of interest.
