# Supplementary material for: Engineering of Humanized Antibodies Against Human Interleukin 5 Receptor Alpha Subunit That Cause Potent Antibody-Dependent Cell-Mediated Cytotoxicity
Source: Front Immunol. 2021 Jan 8;11:593748. doi: 10.3389/fimmu.2020.593748 (PMC7820887; doi:10.3389/fimmu.2020.593748)
Supplement: Supplementary file 1 [file DataSheet_1.pdf]

Supplementary Material

Supplementary Table 1. Blood eosinophil count measured in patients with SEA and healthy controls.

Blood eosinophil count (cells/µL)	Patients with SEA (n=5)	Healthy controls (n=7)
Mean (± SD)	701 (± 392)	220 (± 57)
Median (IQR)	585 (383-1078)	200 (172-296)
Range	313–1306	166–300

Abbreviations: SD, standard deviation; IQR, interquartile range.

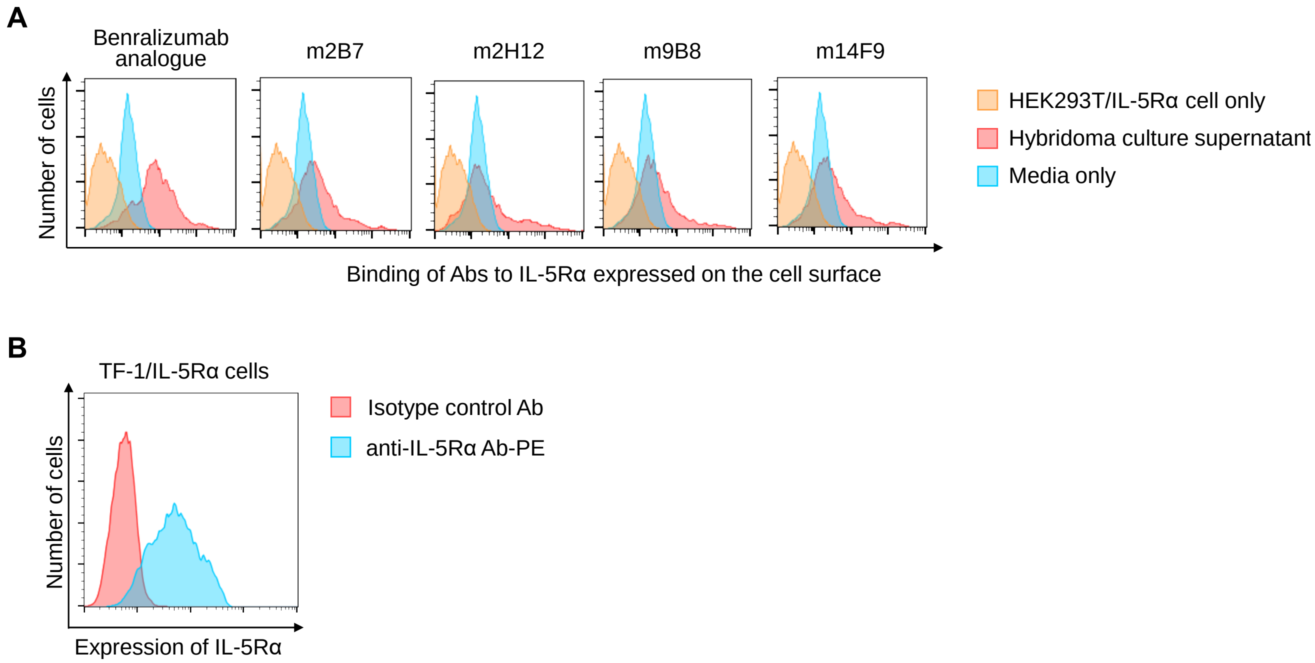

Supplementary Figure 1. Characterization of murine Abs raised against IL-5Rα, and establishment of a stable TF-1/IL-5Rα cell line. (A) Flow-cytometric analysis of binding of the benralizumab analog and hybridoma culture supernatants to the cell surface–expressed IL-5Rα on transiently IL-5Rα–expressing HEK293T (HEK293T/IL-5Rα) cells. The binding of the benralizumab analog and of murine Abs was detected by means of an Alexa 488–conjugated anti-human IgG Ab and an Alexa 488–conjugated anti-mouse IgG Ab, respectively. (B) Flow-cytometric analysis of the cell surface expression of IL-5Rα on the established stable TF-1/IL-5Rα cell line, as determined by labeling with a PE-conjugated Ab against IL-5Rα.

A

	VH-CDR2																
Kabat No.	50	51	52	52a	53	54	55	56	57	58	59	60	61	62	63	64	65
hu2B7	H	I	Y	P	S	E	S	Y	T	N	Y	N	Q	K	F	K	D
5R65	·	·	·	·	N	K	N	E	N	Y	·	·	H	·	·	·	·
5R68	·	·	·	·	T	A	T	I	A	V	·	·	D	·	·	·	·
5R80	·	·	·	·	Q	K	T	L	T	I	·	·	H	·	·	·	·
5R86	·	·	·	·	T	S	S	V	K	F	·	·	N	·	·	·	·

B

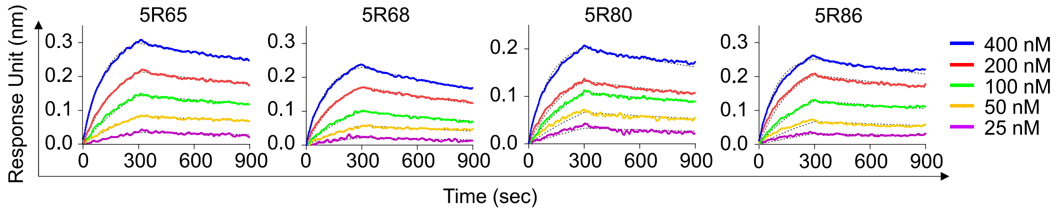

C

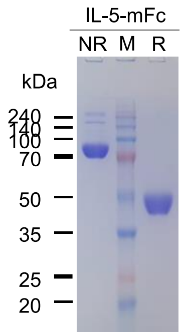

Supplementary Figure 2. Characterization of the isolated anti-IL-5Rα Abs from the VH-CDR2–randomized hu2B7 Ab library. (A) Amino acid sequence alignment of the hu2B7 variants isolated from the hu2B7 Ab library (from Figure 2a). (B) Binding isotherms of the immobilized anti-IL-5Rα Abs toward soluble antigen sIL-5Rα, as measured by bio-layer interferometry. The concentrations of sIL-5Rα are indicated (colored). The kinetic interaction parameters are given in Table 1. (C) Purified IL-5-mFc proteins (3 μg) were analyzed by 12% SDS-PAGE under reducing ("R") or nonreducing ("NR") conditions and then were stained with Coomassie Brilliant Blue.

A

	VH-CDR3												
Kabat No.	95	96	97	98	99	100	100a	100b	100c	100d	100e	101	102
5R65	D	Y	Y	G	R	S	Y	Y	Y	A	M	D	Y
5R65.7	E	F	·	·	·	Q	·	·	Q	·	·	·	·
5R65.10	E	·	·	·	·	·	·	·	A	·	·	·	·
5R65.14	E	H	·	·	·	P	·	·	N	·	·	·	·
5R65.18	E	·	·	·	·	T	·	·	S	·	·	·	·
5R65.39	E	F	·	·	·	·	R	·	S	·	·	·	·
5R65.45	E	·	·	·	·	·	·	·	N	·	·	·	·

B

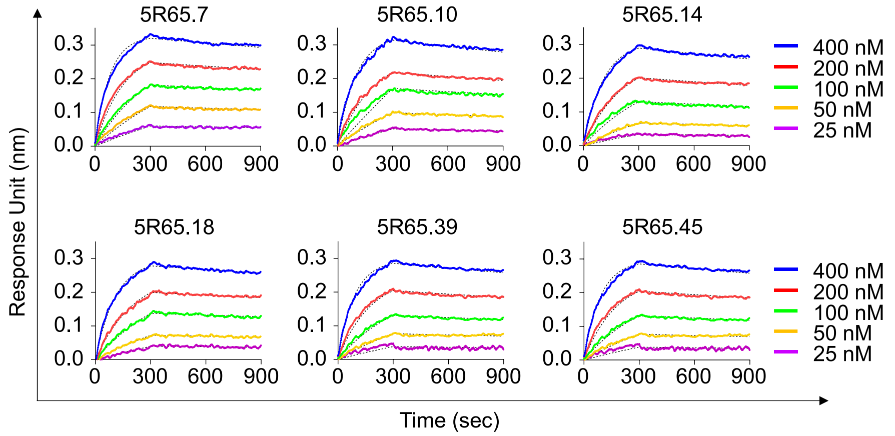

Supplementary Figure 3. Characterization of the isolated anti-IL-5Rα Abs from the VH-CDR3– and VL-CDR3–randomized 5R65 Ab library. (A) Amino acid sequence alignment of 5R65 variants isolated from the 5R65 Ab library (from Figure 3A). (B) Binding isotherms of the immobilized anti-IL-5Rα Abs in relation to sIL-5Rα, as measured by bio-layer interferometry. The concentrations of sIL-5Rα are indicated (colored). The kinetic interaction parameters are listed in Table 1.

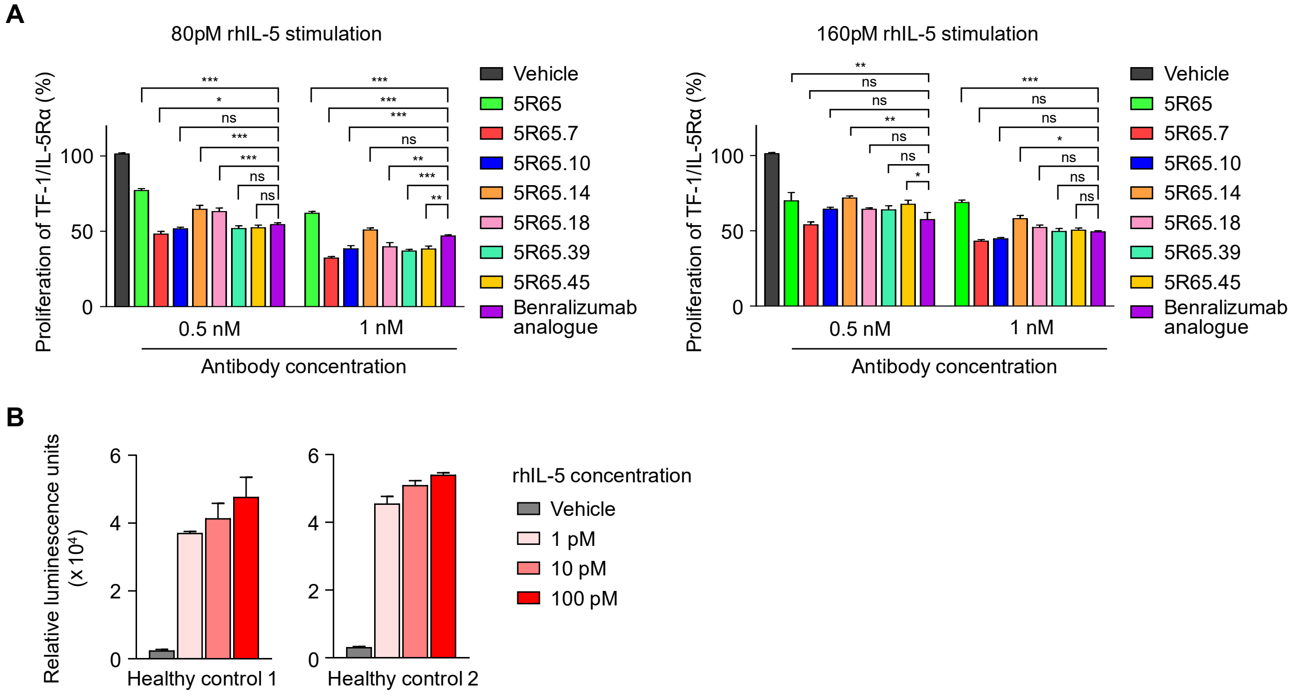

Supplementary Figure 4. (A) Dose-dependent effects of rhIL-5 (80 and 160 pM) on the antagonistic function of the indicated anti-IL-5Rα Abs against the rhIL-5-dependent proliferation of TF-1/IL-5Rα cells. The cells were stimulated for 40 h with either 80 pM or 160 pM of rhIL-5 in the presence of the Abs (0.5 and 1 nM), prior to the cell proliferation assay. Data are presented as a percentage (mean ± SD) of the proliferation relative to PBS-treated samples (vehicle). Significance was tested by two-way ANOVA followed by the Newman–Keuls *post hoc* test. *, $P < 0.05$; **, $P < 0.01$; ***, $P < 0.001$; ns, not significant versus the benralizumab analogue–treated group.

(B) Dose-dependent effects of rhIL-5 (1, 10, and 100 pM) on the proliferation of eosinophils from healthy controls. The proliferation of eosinophils is represented as luminescence units (mean ± SD), measured by CTG assay after stimulation with the indicated concentrations of rhIL-5 for 40 h.

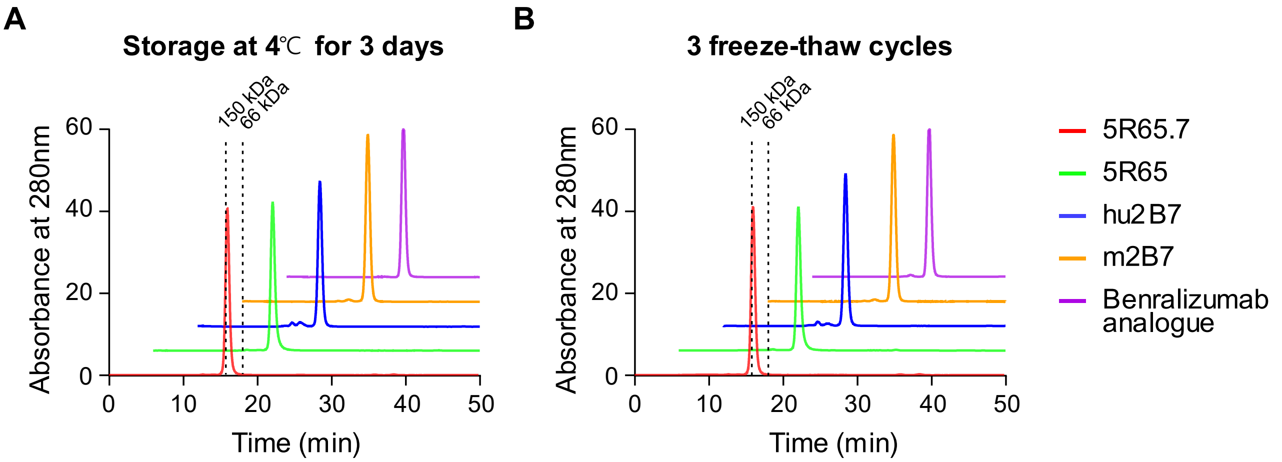

Supplementary Figure 5. Size exclusion elution profiles of Abs stored at 4°C for 3 days (A) and exposed to 3 freeze-thaw cycles (B), monitored at 280 nm. In (B), the anti–IL-5Rα Abs (3 mg/mL in PBS buffer, pH 7.4) were exposed to 3 freeze-thaw cycles of freezing at -80°C for 30 min and then thawing at room temperature. Then the samples (20 μL, 1 mg/mL) were injected onto SEC column to detect any soluble aggregates and degraded fragments. Abs stored at 4°C for 3 days were included as a control. Two independent analyses were performed with the same results. The dotted lines indicate the elution positions of molecular weight standards.

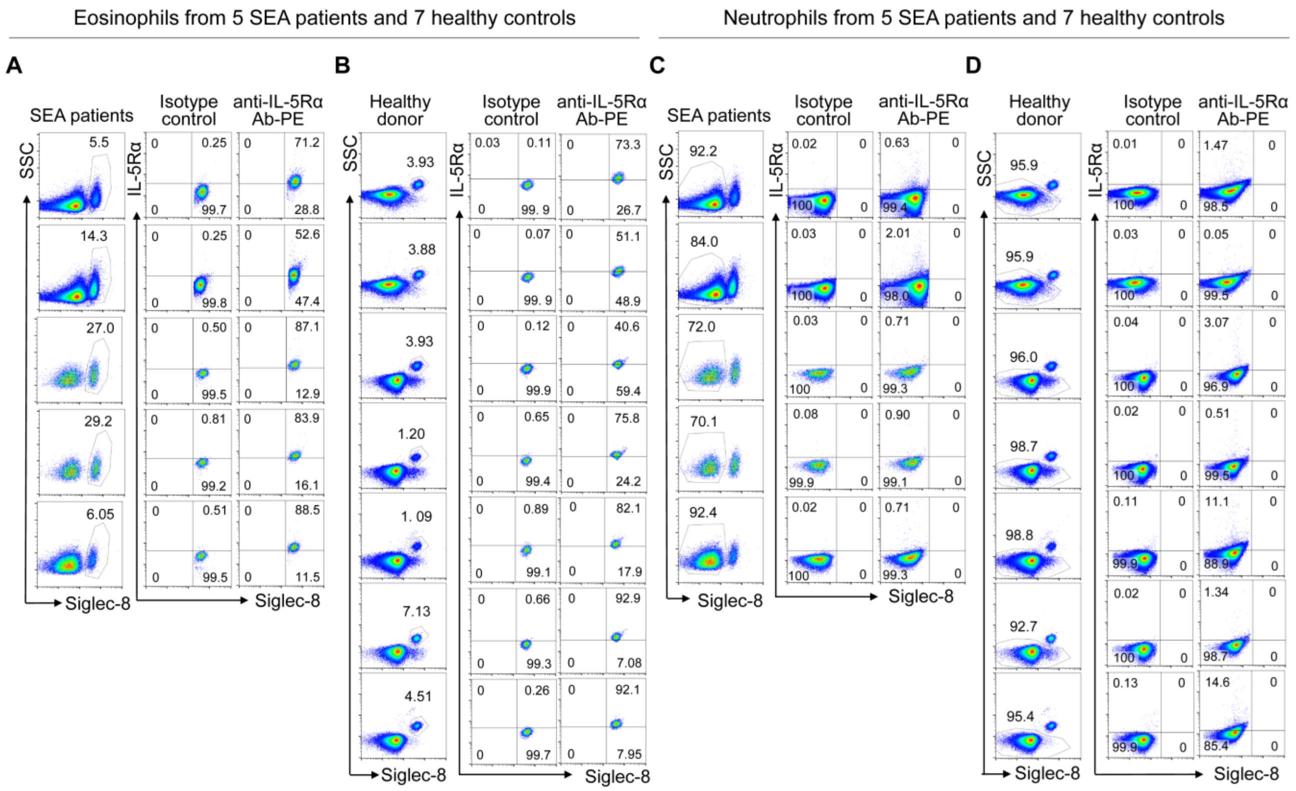

Supplementary Figure 6. Isolation and characterization of eosinophils and neutrophils from seven healthy controls and five patients with SEA. (A–D) Left panel: Flow-cytometric sorting gate plots of the granulocyte fraction of peripheral blood of patients with SEA (n = 5) and individual healthy controls (n = 7); the fraction was enriched by Ficoll-Paque separation and then stained with the anti–Siglec-8 Ab. According to side scatter characteristics (SSC) and the cell surface marker Siglec-8, Siglec-8$^+$ and Siglec-8$^-$ cells were defined as eosinophils (A, B) and neutrophils (C, D), respectively. Right panel: Dot plots of eosinophils (A, B) and neutrophils (C, D) sorted from the indicated gates and then costained with an allophycocyanin-conjugated Ab against Siglec-8 and a PE-conjugated Ab against IL-5Rα or an isotype control Ab.
